# Current understanding of the impact of United States military airborne hazards and burn pit exposures on respiratory health

**DOI:** 10.1186/s12989-024-00606-5

**Published:** 2024-10-21

**Authors:** Janeen H. Trembley, Paul Barach, Julie M. Tomáška, Jedidah T. Poole, Pamela K. Ginex, Robert F. Miller, Jacob B. Lindheimer, Anthony M. Szema, Kimberly Gandy, Trishul Siddharthan, Jason P. Kirkness, Joshua P. Nixon, Rosie Lopez Torres, Mark A. Klein, Timothy R. Nurkiewicz, Tammy A. Butterick

**Affiliations:** 1 Minneapolis Veterans Affairs Health Care System, Minneapolis, MN USA; 2 Department of Laboratory Medicine and Pathology, University of Minnesota, Minneapolis, MN USA; 3 Masonic Cancer Center, University of Minnesota, Minneapolis, MN USA; 4 Thomas Jefferson University, Philadelphia, PA USA; 5 Burn Pits 360, Robstown, TX USA; 6 School of Public Health and Tropical Medicine, Tulane University, New Orleans, LA USA; 7 School of Nursing, Stony Brook University, Stony Brook, NY USA; 8 Department of Medicine, Vanderbilt University, Nashville, TN USA; 9 James A. Haley Veterans Hospital, Tampa, FL USA; 10 Three Village Allergy and Asthma, PLLC, South Setauket, NY USA; 11 Department of Occupational Medicine, Epidemiology, and Prevention, Donald and Barbara Zucker School of Medicine at Hofstra/Northwell, Hempstead, NY USA; 12 Division of Allergy and Immunology, Donald and Barbara Zucker School of Medicine at Hofstra/Northwell, Hempstead, NY USA; 13 Division of Pulmonary and Critical Care, Donald and Barbara Zucker School of Medicine at Hofstra/Northwell, Hempstead, NY USA; 14 Play-It-Health, Overland Park, KS USA; 15 Division of Pulmonary and Critical Care Medicine, University of Miami, Miami, FL USA; 16 4DMedical, Los Angeles, CA USA; 17 Department of Surgery, University of Minnesota, Minneapolis, MN USA; 18 Department of Medicine, University of Minnesota, Minneapolis, MN USA; 19 Department of Physiology, Pharmacology and Toxicology, West Virginia University School of Medicine, Morgantown, WV USA; 20 Center for Inhalation Toxicology (iTOX), West Virginia University School of Medicine, Morgantown, WV USA; 21 Department of Food Science and Nutrition, University of Minnesota, St Paul, MN USA; 22 Department of Neuroscience, University of Minnesota, Minneapolis, MN USA

**Keywords:** PACT Act, Veterans, Military, Burn pit, Airborne hazards, Inhalation, Burn Pits 360, Pulmonary, Inflammation, Implementation

## Abstract

Millions of United States (U.S.) troops deployed to the Middle East and Southwest Asia were exposed to toxic airborne hazards and/or open-air burn pits. Burn pit emissions contain particulate matter combined with toxic gasses and heavy metals. Ongoing research has demonstrated that exposures to the airborne hazards from military burn pits have profound and lasting health and wellness consequences. Research on the long-term health consequences of exposure to open burn pits has been limited. Work continues to understand the scope of the health impacts and the underlying pathobiology following exposures and to establish care standards. The U.S. Sergeant First Class Heath Robinson Honoring our Promise to Address Comprehensive Toxics (PACT) Act was signed into law August 2022. This act expands the benefits and services to U.S. Veterans exposed to toxicants, requires the Veterans Health Administration to provide toxic exposure screening, and supports increased research, education, and treatment due to toxic occupational exposures. This review highlights the state of the science related to military burn pit exposures research with an emphasis on pulmonary health. Clinical data demonstrate areas of reduced or delayed pulmonary ventilation and lung pathologies such as small airways scarring, diffuse collagen deposition and focal areas of ossification. Identification and characterization of foreign matter deposition in lung tissues are reported, including particulate matter, silica, titanium oxides, and polycyclic aromatic hydrocarbons. These data are consistent with toxic exposures and with the symptoms reported by post-deployment Veterans despite near-normal non-invasive pulmonary evaluations. On-going work toward new methods for non-invasive pulmonary diagnoses and disease monitoring are described. We propose various studies and databases as resources for clinical and health outcomes research. Pre-clinical research using different burn pit modeling approaches are summarized, including oropharyngeal aspiration, intranasal inhalation, and whole-body exposure chamber inhalation. These studies focus on the impacts of specific toxic substances as well as the effects of short-term and sustained insults over time on the pulmonary systems.

## Background

**Fig. 1:**
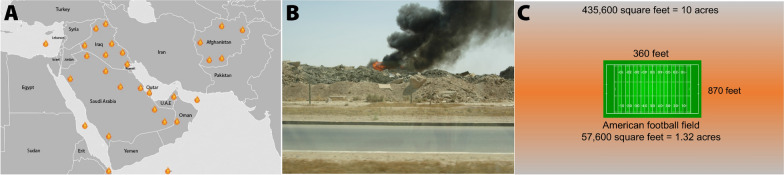
U.S. Military Burn Pit Locations in the Middle East and Southwest Asia. The images illustrate the environmental and health hazards posed by burn pits, highlighting the scale of smoke and airborne toxicants released and providing context for discussions on toxic exposures and their potential health impacts on military personnel. **A**, Regions and countries where burn pits were located. The fire icons indicate the approximate locations of U.S. military sites with burn pits. **B**, Burn pit emissions at Balad Air Base, Iraq. Photos of the Balad Burn Pit by Dr. Julie Tomáška (Ret. Air National Guard) in 2005, with permission. **C**, The Balad Air Base burn pit was one of the largest documented and equivalent to 7.6 American football fields. For reference, one football field is 5352 m^2^, and the burn pit size was approximately 40,466 m^2^. Images adapted from Perveen et al. 2023 [5]

Millions of U.S. military Veterans who served during the Gulf War era and in post-9/11 deployments were frequently and repeatedly exposed to a variety of airborne hazards, including particulate matter (PM) from desert dust, diesel fumes, and emissions from open-air burn pits. Open burn pits, prevalent on military bases across the Middle East and Southwest Asia, were the primary means for disposing of military, industrial, and medical waste. The Department of Defense (DoD) estimated that the larger bases collectively incinerated up to 85,000 pounds of daily waste [1, 2]. These facilities were a common feature, with 30–90% of military sites in Iraq and Afghanistan utilizing burn pits [3]. The most extensive of these were located at U.S. Joint Base Balad, Iraq, and covered more than 25 acres, illustrating the exposure scale and risks of these operations. Often situated near military housing, work areas, and dining facilities, the burn pits posed significant health risks to U.S. soldiers due to the complex mixtures of gases and particulate matter released [1]. The exposure to these toxic emissions has been linked to a wide range of pulmonary and extra-pulmonary morbidities, as well as various physical and mental health symptoms observed in Veterans after their deployments [4], Fig. 1.

A broad range of waste was burned and included computers, animal carcasses, medical waste, lithium ion batteries, plastic waste, Styrofoam, insecticide canisters, DEET-soaked items, human excrement, food waste, and vehicles [6]. A commonality to burn pits was the regular use of fuels such as jet propellant-8 (JP-8) and/or jet fuel with military additives (JAA) as accelerants [7]. This waste disposal method produces very high airborne concentrations of mixed toxic emissions such as volatile organic compounds and metals. Burn pit smoke contains particulate matter ranging from 40 to 120 µg/m^3^, with average concentrations well above U.S. air pollution standards [8, 9]. Toxicants identified in burn pit emissions include polycyclic aromatic hydrocarbons (PAHs), polychlorinated dibenzodioxins, furans, carbon- and silica-based particulate matter, volatile organic compounds (VOCs), carbon monoxide, ash, formaldehyde, hydrogen cyanide, nitrogen dioxide, sulfur dioxide, and heavy metals [10]. Many of these are known carcinogens, neurotoxins, and endocrine disruptors linked to chronic illnesses and deaths [10–14]. Concentrations of these toxicants measured near military burn pits often exceeded U.S. EPA air quality standards by substantial margins. For instance, benzene levels reached 0.07 ppm, over 20 times higher than the EPA's acceptable long-term exposure of 0.003 ppm [10]. Levels of benzo[a]pyrene, a carcinogenic PAH, were 10 to 100 times above the EPA's guideline of 0.00012 µg/m^3^ [10]. Heavy metals like lead were found at concentrations far exceeding the EPA's Reviewing National Ambient Air Quality Standards (NAAQS) of 0.15 µg/m^3^, indicating significant health risks for exposed military personnel [10].

**Fig. 2:**
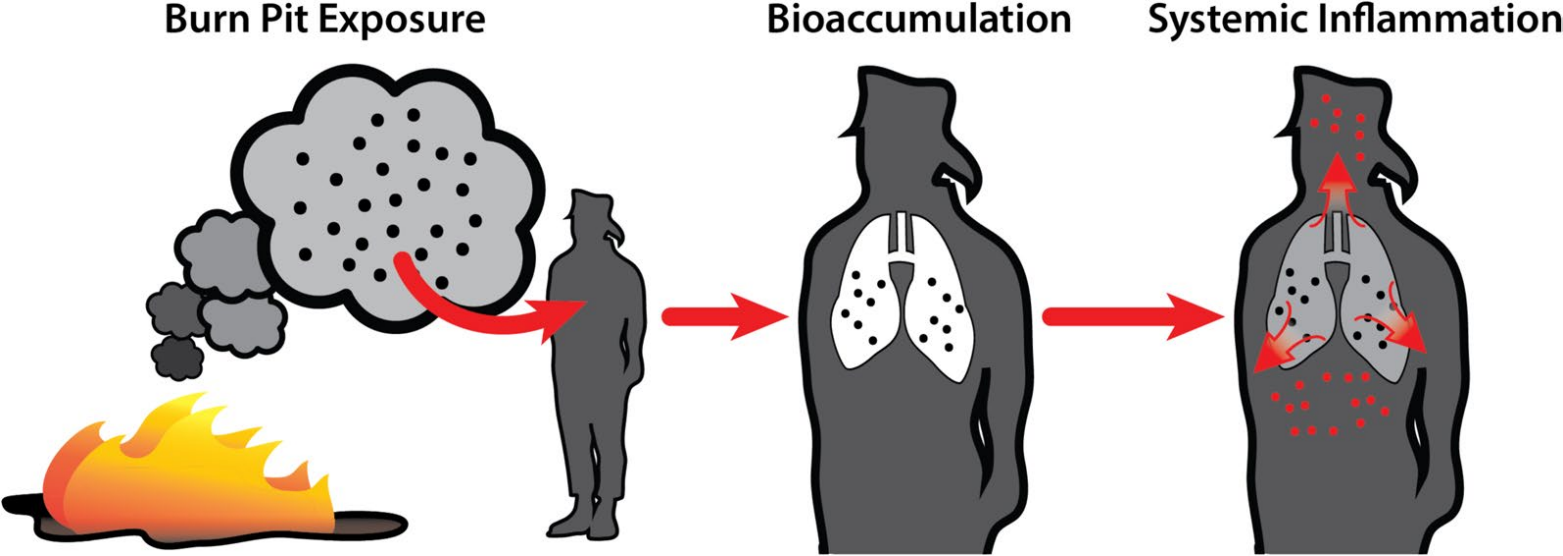
Conceptual diagram of military burn pit exposure health effects. PM and toxins are generated by incomplete combustion in burn pits. A portion of PM becomes airborne and is inhaled by personnel in the area. Inhaled PM enters the lungs where accumulation occurs coincident with activation of systemic inflammatory response. Exposure to airborne toxicants from burn pits can lead to the bioaccumulation of PM and heavy metals in the body. These toxic substances accumulate over time in various organs and tissues, creating a reservoir of harmful compounds with prolonged adverse effects [15]. Bioaccumulation contributes to systemic inflammation, a chronic immune response marked by elevated inflammatory cytokines that can affect multiple organ systems. Systemic inflammation has been identified as a key factor in the development of DRRDs, such as bronchial asthma, constrictive bronchiolitis, and interstitial lung disease. Persistent inflammation can damage lung tissues, promote fibrosis, and impair respiratory function. Moreover, chronic systemic inflammation, combined with carcinogenic substances like PAHs, can increase the risk of lung cancer by causing genetic mutations, promoting cellular proliferation, and inhibiting apoptosis [25]

Data on the impacts of burn pit exposures on lung health continues to emerge, and across scientific communities a better understanding is needed. Although measurable changes in pulmonary function and disease diagnosis can be linked to burn pit exposure, they have not been consistently conclusive [9, 15–22]. Clinical findings from multiple studies reveal a complex array of respiratory issues among Veterans described as “Deployment Related Respiratory Disease” (DRRD) [16], Fig. 2). This term underscores the broad range of pulmonary pathologies associated with military burn pits. One DRRD condition is constrictive bronchiolitis, rare in healthy young adults but prevalent among Veterans exposed to burn pit emissions [18, 20]. Several DRRD conditions are characterized by irreversible airflow limitations due to small airway fibrosis. However, the literature also presents conflicting findings. Some studies confirm specific pathologies like constrictive bronchiolitis related to exposures, while others struggle to directly tie burn pit exposures to chronic respiratory diseases, highlighting the challenges with inconsistent epidemiological associations and underscoring the need for more precise exposure assessment tools and long-term health tracking [23, 24]. The etiology of many respiratory symptoms remains unresolved, underscoring the need for improved research methodologies and enhanced clinical surveillance to conclusively determine the causal relationships associated with these toxicants and to advance Veteran care.

There is evidence of respiratory health impacts among military personnel from other countries exposed to similar occupational and environmental conditions [1]. Data also suggest increased respiratory symptoms among Polish soldiers, while Russian soldiers reported more respiratory issues linked to their deployments in Afghanistan before 2001 [1–4]. A study of Swedish military personnel who served in Afghanistan in years 2008 to 2009 found a higher prevalence of respiratory symptoms including wheezes, nocturnal coughing, and chronic bronchitis as compared to a civilian group [6]. A significant link was observed between time spent in desert environments and symptoms such as wheezing with and without breathlessness. Exposure to dust storms was associated with nocturnal cough and chronic bronchitis. However, there is limited data on post-deployment follow-up of these groups [1].

The passage of the 2022 Sergeant First Class (SFC) 118 Heath Robinson Honoring Our Promise to Address Comprehensive Toxics (PACT) Act has broadened the Department of Veterans Affairs (VA) healthcare scope, such as inclusion of presumptive illnesses and enhanced research pertaining to burn pit exposures [26–28]. Systematically gathering both pre-clinical and clinical data and clinical outcomes information, ensures that research findings can be integrated into healthcare practices. This review aims to summarize the current state of scientific knowledge regarding research on military burn pit exposures, with a particular focus on pulmonary health. We discuss the challenges associated with clinical diagnoses in affected individuals, explores the potential benefits of ongoing clinical studies, and examines the role of technological advancements and pre-clinical models in understanding and mitigating burn pit exposures.

### Clinical knowledge—pulmonary findings

In 2004, Fort Campbell clinicians reported that multiple soldiers were returning from Operation Iraqi Freedom with unexplained shortness of breath [20]. All were physically fit at the time of deployment, but unable to complete a routine two-mile run within regulation time on return post deployment. Standard clinical evaluations, including x-rays, pulmonary function testing, high resolution CT scans and cardiac screening, failed to explain their symptoms. Vanderbilt University clinicians were asked to help evaluate this cohort and were similarly perplexed by the large number of symptomatic deployers with normal non-invasive testing results. Surgical lung biopsies demonstrated pathologic changes consistent with toxic inhalation [20]. Small airway scarring known as constrictive bronchiolitis was the most striking pathologic finding and all lung compartments had features of toxic lung injury [20]. Furthermore, lung morphometry demonstrated a more generalized tissue reaction with diffuse collagen deposition in almost all lung compartments [18]. Others at National Jewish Health and the University of Michigan have reported similarly symptomatic deployers with normal or near normal non-invasive testing and inhalational injury on biopsy; deployment-related lung histopathology characteristics, especially in the small airways of the lower lobes, are emerging [4, 9, 29].

A 2022 Department of Veterans Affairs (VA) modified Delphi panel, comprising basic scientists and clinicians, reviewed key issues related to constrictive bronchiolitis and recognized several important findings. The panel highlighted the high prevalence of unexplained shortness of breath among deployed Veterans, the limitations of non-invasive testing in effectively characterizing lung injuries, and the evidence of diffuse lung injury extending beyond small airway pathology [16]. The panel applied the designation of DRRD to this group of symptomatic Veterans. This decision acknowledged constrictive bronchiolitis did not adequately characterize the extent of emerging pathologic findings but reflects a poorly defined set of heterogenous respiratory syndromes with clear physiological impacts. DRRD provided a designation which would permit assessment of symptomatic Veterans without requiring invasive biopsies. The PACT Act included constrictive bronchiolitis as well as twenty-two other disorders as being presumptively linked to deployment. Veterans with unexplained shortness of breath, and more specifically, those with biopsy-proven constrictive bronchiolitis continue to experience challenges in procuring medical evaluations.

Veterans with constrictive bronchiolitis continue to experience challenges in accessing expert medical evaluations. To date, approximately 350 lung biopsies have been conducted on previously deployed individuals to investigate respiratory symptoms and potential lung injuries [9, 10]. Some studies have also utilized advanced imaging techniques and pulmonary function testing to assess airway abnormalities in symptomatic military personnel [16, 30]. A recent study performed elemental analysis of lung tissue comparing post-9/11 military personnel with distal lung disease to control lung tissue samples. Significantly higher levels of silica, titanium oxides, and aluminosilicates and other silicates were found using inductively coupled plasma mass spectrometry in the lung tissue of the deployed personnel [30]. A different study analyzed biopsies from burn pit-exposed Veterans with constrictive bronchiolitis and severe pulmonary fibrosis, along with age-matched, non-deployed controls, for foreign materials and pathological characteristics [22]. The lung biopsies contained not only particulate dust from the burn pits but also polycyclic aromatic hydrocarbons, burned JP-8 (jet propellant-8) fuel, and metals. These metals include burned or oxidized titanium, known carcinogens which cause inflammation and fibrosis. Burned metals are consistent with the higher temperature of burn pits and were not found in control tissues. Focal areas of ossification, a phenomenon known to be generated from the alkaline properties of the dust in other model systems, were also detected.

This group further demonstrated pulmonary damage associated with exposures to environmental hazards and burn pits in thirty-one previously healthy Veterans serving in Iraq and Afghanistan who self-reported exposure to airborne hazards including new onset dyspnea [22]. Over 60% of the Veterans tested were found on impulse oscillometry and maximal expiratory pressures to have hyper-responsive airways demonstrated by abnormal airway reactance. Peripheral and distal airway resistance were also increased. These findings were accompanied by decreased respiratory muscle strength as measured by reductions in maximum expiratory pressures. These findings demonstrate that Veterans exposed to burn pits are at risk for pulmonary fibrosis, reduction of respiratory muscle strength, distal airway narrowing, and hyper-responsiveness.

Military airborne hazards and exposures-related lung diseases such as constrictive bronchiolitis are challenging pulmonary conditions to diagnose, often presenting with nonspecific respiratory symptoms that can elude detection through conventional imaging and pulmonary function tests [18].The introduction of functional imaging using velocimetry marks an advancement in the ability to assess and understand this condition. Velocimetry allows the display of structural imaging with dynamic functional assessment, thus enabling clinicians to visualize and quantify the regional distribution of lung ventilation. This technology is particularly adept at identifying areas of impaired ventilation and airflow limitation, hallmarks of constrictive bronchiolitis, which may not be apparent in standard imaging modalities.

The application of this technology in clinical research settings allows to differentiate constrictive bronchiolitis from other respiratory conditions that present with similar clinical manifestations. In patients with biopsy-confirmed constrictive bronchiolitis, functional imaging using velocimetry provided a detailed map of lung ventilation [31]. This allowed for the identification of areas with reduced or delayed ventilation, which often corresponded to the histopathological findings of airway constriction. By comparing the ventilation profiles of these patients to those of healthy individuals or patients with other pulmonary conditions, clinicians can gain a deeper understanding of the disease's dynamic impact on lung function. This is particularly crucial in cases where Veterans exhibit symptoms such as unexplained dyspnea but have normal findings on traditional pulmonary function tests and chest CT scans. The ability to quantitatively assess changes in lung ventilation may guide treatment decisions and improve patient outcomes.

### Future clinical applications, approaches and resources

Challenges continue in the diagnosis and monitoring of Veterans with respiratory symptoms post-deployment. Research is underway to establish non-invasive and sensitive clinical tests to address these challenges in small airways diseases. Velocimetry is instrumental in the quantitative analysis of lung imaging, both structural and functional [32]. In pre-clinical studies, velocimetry-based lung imaging technology has contributed to our understanding of pulmonary ventilation dynamics [32–34]. Velocimetry allows for detailed comparisons between normal lung function and pathological conditions, as observed in animal injury models [35], and enables the ability to detect slow-filling zones of the lung, suggesting airway constriction. Velocimetry aids in a detailed understanding of lung ventilation distribution, using standard imaging equipment found in clinical settings [36, 37]. The technology has been effective in revealing specific pathological characteristics in patients with DRRDs, which often present with shortness of breath despite normal pulmonary function tests and inconclusive chest CT scans [38]. Further non-invasive technologies are under investigation in Veterans to assess pulmonary function. Forced oscillation testing or impulse oscillometry uses sound waves to measure the amount of resistance to the normal movement of air in and out of the lungs [39–41]. Multiple breath washout or lung clearance index testing measures “wash out” and replacement of pure oxygen with room air or lung clearance of an inert gas such as nitrogen [42, 43]. Finally, quantitative chest computed tomography (CT) uses high resolution X-ray-generated CT scans and computational analytics to construct detailed images of the lung and identify and quantify small airway abnormalities [44].

Symptoms rarely occur in isolation and tend to ‘cluster’ together. For example, a Veteran may present clinically with a cluster of respiratory symptoms such as shortness of breath, cough, and fatigue frequently occurring simultaneously. Health outcomes research is important in understanding how best to plan, treat, follow up and support Veterans with military exposures. Research studies and national initiatives have identified a wide range of symptoms and health issues in active-duty military and Veteran service members following deployment. The reported symptoms involve virtually all body systems including cardiovascular, respiratory, musculoskeletal, urogenital, mental health, and others. It is important to note that these Veterans were young (average age 33) and healthy prior to deployment, and self-reported symptoms increased significantly post-deployment [45, 46]. While we know that Veterans report a wide range of symptoms post-deployment, we do not yet know which symptoms tend to cluster together, which are most severe or distressing, and what is the lived experience of Veterans whose health has changed so dramatically.

Military burn pit exposures, similar to general air pollution exposures, can induce systemic effects impacting multiple organ systems, including cardiovascular, respiratory, and neurological systems [47]. Similarly, burn pit emissions contain a mix of toxicants such as PAHs, VOCs, heavy metals, and dioxins, and have been linked to comparable systemic effects and adverse health outcomes in Veterans [11]. While both types of exposures share common etiological pathways of injury, the composition of toxicants in burn pit smoke can lead to unique and devastating disease profiles. Burn pits often burn diverse waste materials, including medical and electronic waste, introducing toxicants not typically found in urban air pollution [11]. This difference likely leads to distinct conditions, such as constrictive bronchiolitis, which are uncommon in non-military populations. Although there is overlap in the health impacts, specific factors in burn pit emissions require further study to understand their unique long term effects.

Two data registries are currently collecting exposure and health data from Post-9/11 deployed personnel: the Airborne Hazards and Open Burn Pit Registry, established by the VA, and the Burn Pits 360 registry, established by a Veterans advocacy non-profit organization [48, 49]. In addition, there are several active nationwide studies. The VA Cooperative Studies Program #595, Pulmonary Health and Deployment to Southwest Asia and Afghanistan (SHADE; NCT02825654) is being conducted at multiple VA medical centers and has planned enrollment of 6200 Veterans [50]. This large study is designed to evaluate cumulative exposure to PM_2.5_ in relation to lower lung function and respiratory health. A novel aspect of SHADE is that a spatial–temporal exposure grid for PM_2.5_ levels at deployment locations will be built using NASA satellite and military airport visibility records and linked with enrollee’s locations and exposure duration history [51]. The Millennium Cohort Study has enrolled more than 250,000 U.S. service members since 2001. This study tracks how military occupational exposures continue to affect the long-term health of U.S. service members [52]. Respiratory health outcomes data from these and other on-going studies were compiled and described in a 2020 report from the National Academies of Sciences, Engineering, and Medicine [53].

Veterans Health Administration (VHA) Research Enterprise resources facilitate military exposures research for VA and collaborative researchers. These include data-related resources such as the Million Veteran Program (MVP), the Centralized Interactive Phenomics Resource (CIPHER), the Center for Data & Computational Science (C-DACS), and the VA Informatics and Computing Infrastructure (VINCI) [52, 54–56]. Biorepositories for military exposures research include VA Science and Health Initiative to Combat Infectious and Life-Threatening Diseases (VA SHIELD) and the Department of Defense serum Repository [13, 57]. Mechanisms for facilitating non-VA funded research include the VA Partnered Research Program and National Association of Veterans’ Research and Education Foundations (NAVREF) [58]. A recent research initiative at the VA is the “Military Exposures Research Program,” which aims to advance research on military exposure assessments and to better understand the long-term effects of military exposures on Veterans’ health outcomes, ultimately informing healthcare and policy decisions.

Over the last several years, VA has made a significant investment in precision medicine. The VA Precision Medicine and Oncology Programs have the transformative potential to benefit Veterans seeking care for possible burn pit exposure-related malignancies. Molecular testing, including DNA sequencing, RNA sequencing, and pharmacogenomics, amongst others, has rapidly become standard of care in oncology. These technologies greatly enhance the prediction of efficacy of various medications based on the molecular testing and improve accuracy of prognosis prediction. To date, application of such technology in the study of military exposures has been modest; however, the Million Veteran Program, the first of its kind study, has accrued molecular and other data on over 1 million study participants, resulting in numerous publications related to genomics and disease outcomes, and providing an invaluable resource for assessing the risk and pathobiology of disease from airborne toxins [59–61]. There is a lack of genomic and other molecular information to form the basis of precision medicine-based clinical trials for patients afflicted with diseases at least partially resulting from military exposures. Leveraging VA and other precision medicine studies to facilitate the development of new molecularly-targeted treatments utilizing molecular and genomic techniques is a crucial research strategy for the future.

### Pre-clinical models of burn pit toxicant exposures

Veterans exposed to burn pit emissions were universally subjected to inhalation hazards produced by incomplete combustion. A common characteristic among all burn pits is that they cyclically smolder rather than burn evenly [62]. This constant cycle of smoldering and incomplete combustion of incinerated waste generates emissions that are harmful to human health [63, 64]. Ongoing research supports that increased mixture complexity associates with increased risk of DRRD and extra-pulmonary diseases [16, 45]. Development of preclinical rodent models is central to simulating human exposure to burn pit emissions. The scope of materials incinerated in burn pits is enormous, and it is difficult, if not impossible, to recreate in the laboratory the full range of emissions generated across the Southwest Asia and Middle East Theater of Operations. However, many common substances were regularly combusted in burn pits. These materials include jet fuel, oil, wood, paper, plastics, nylon and rubber [11, 30, 65, 66]. A range of particle sizes are produced by burning these substances, including coarse, fine and ultrafine particulate matter (PM_10_, PM_2.5_ and PM_0.1_, respectively) and PAHs and VOCs are generated as a byproduct of the burned waste [11, 30, 65, 66].

An immediate rate limiting step in translational burn pit exposure research is the challenge of modeling inhalation exposures due to diverse toxicant mixtures. Ideally this modeling includes incomplete combustion of input materials and delivery of emissions in real time to freely moving rodents [67]. Some studies used different materials typical of burn pits such as plywood, cardboard, plastics, and fuels to generate emissions, aiming to replicate the complex chemical exposures experienced in field conditions [62, 65, 68]. Other models have used components such as PM_2.5_, carbon black or dust from Iraqi burn pits to represent PM that were characteristic of emissions [65, 67, 69–71]. These models may not incorporate the full spectrum of materials from open air burn pits, such as biological waste and electronics, potentially limiting their full applicability in predicting human exposures, injuries and long term health outcomes.

**Fig. 3:**
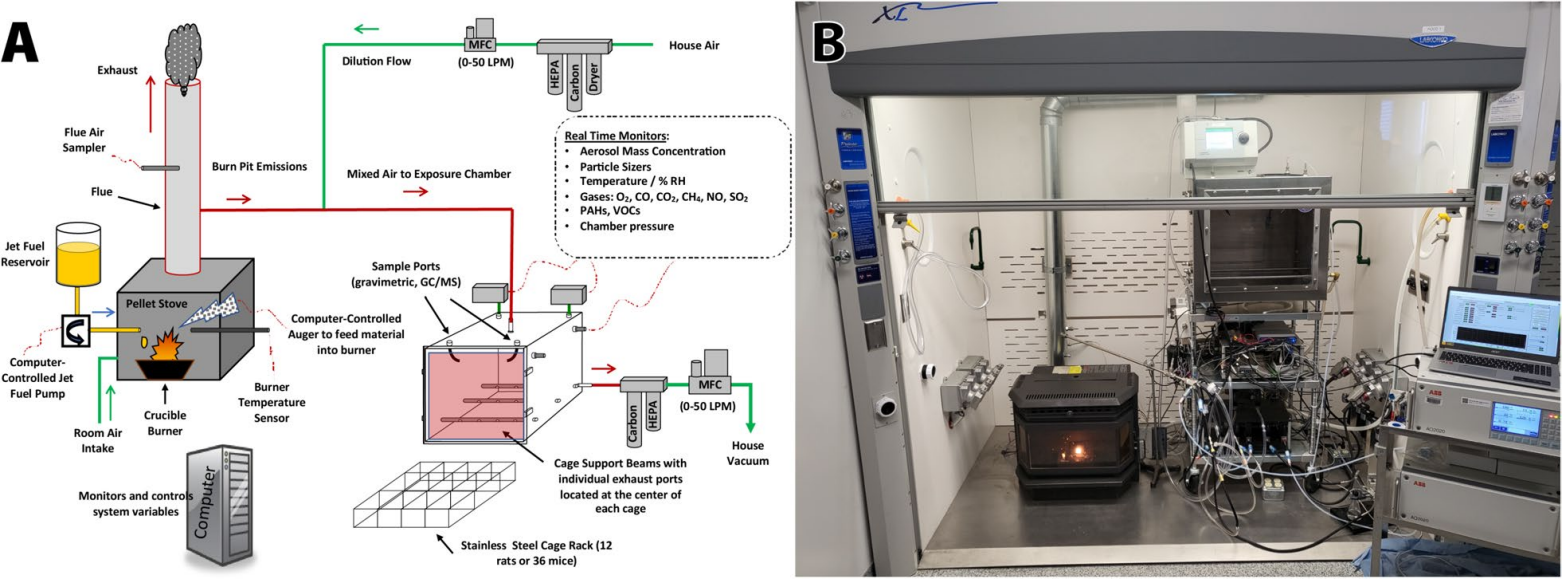
Burn pit simulator and rodent whole body inhalation exposure chamber system. **A,** iTOX Burn Pit Surrogate Generator schematic. Custom mixed pellets are used to feed fuel material into burner. Because of the modular nature, and high air flow volume, high resolution control and air sampling are possible in the burn generator and exposure chamber. HEPA: high efficiency particulate air filer; MFC: mass flow controller; LPM: liters per minute; VOC: volatile organic compound; PAH: polyaromatic hydrocarbon, GC/MS: gas chromatography/mass spectrometry. Exhaust is filtered prior to release above the Health Sciences Center roof. The entire system is enclosed in a walk-in safety hood. **B**, Military burn pit surrogate generator (left) and aerosol exposure system which can house rodent cages (right) contained in the WVU iTOX Inhalation Facility

**Fig. 4:**
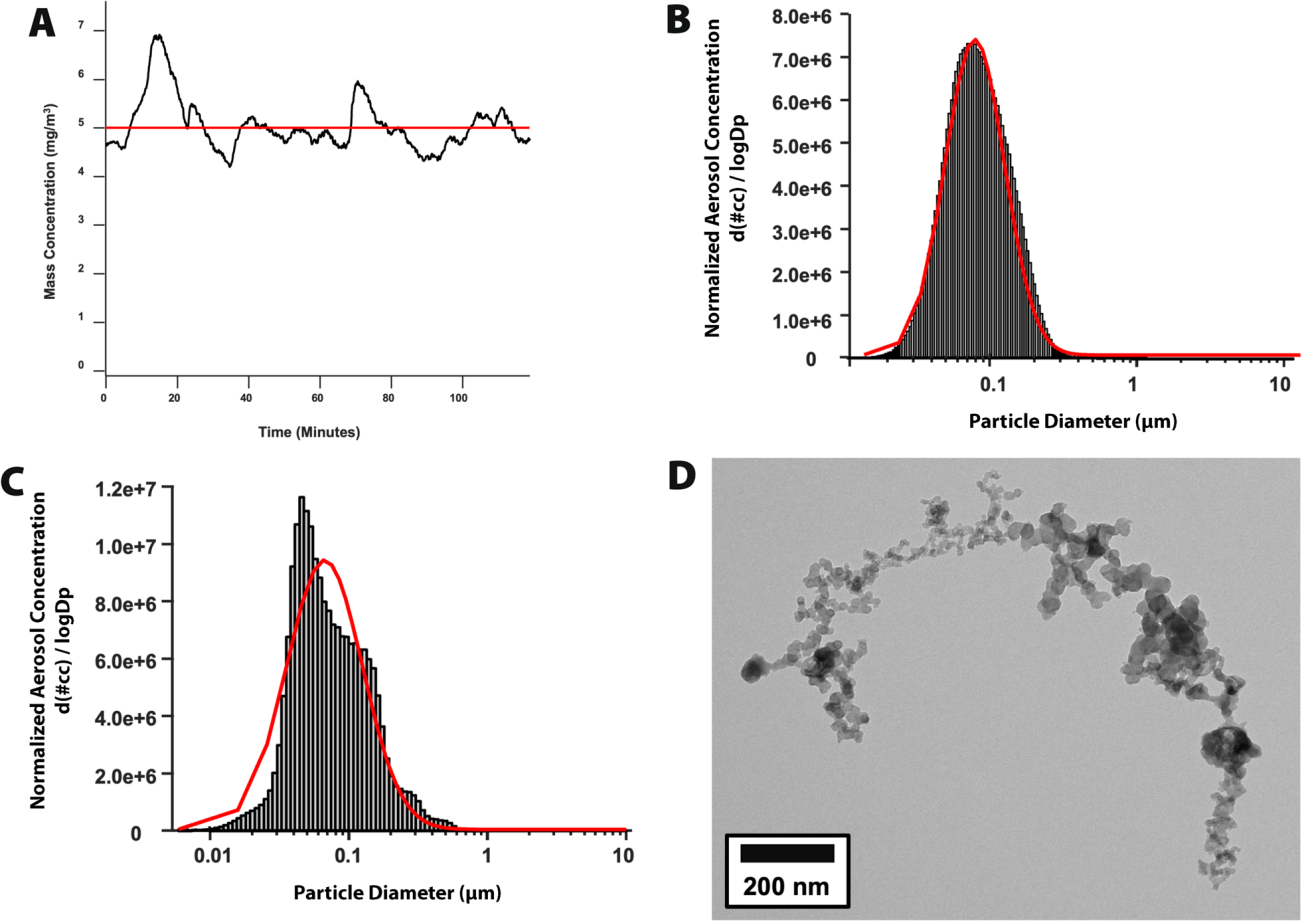
Characterization of emissions from rodent exposure chambers generated in the WVU iTOX burn pit simulator. Representative aerosol profile made in real-time in the exposure chamber produced by combustion of mixed wood pellets, plexiglass and JAA in the combustion chamber. **A**, Particle concentration. **B,** Size distribution determined by scanning mobility particle sizer (SMPS) and aerodynamic particle sizer (APS). **C**, Size distribution determined by high resolution electrical low-pressure impactor (ELPI +).** D**, Transmission electron microscopy image of representative PM in emissions. Lower left, size bar

Air and soil samples from total suspended particle (TSP) measurements collected at multiple military sites and surrounding areas across the Middle East and Southwest Asia over a one-year period revealed elevated levels of PM_10_ and PM_2.5_ [72]. This concentration had a PM_10_ component of 298 µg/m^3^, and a PM_2.5_ component of 111 µg/m^3^, both of which exceed the 24 h Military Exposure Guidelines (250 µg/m^3^ and 65 µg/m^3^, respectively), and were frequently exceeded at multiple other military bases [72, 73]. Operating conditions at concentrations in the mg/m^3^ range would be associated with a closer proximity to the burn pit. Biologic effects have been reported after burn pit smoke exposures at these concentrations [62]. There are different methods used to achieve inhalation delivery of these various toxicants in rodent models, including intranasal inoculation or single instillation, oropharyngeal aspiration, and whole body inhalation within a chamber. Pilot studies carried out at the Center of Inhalation Toxicology (iTox) at West Virginia University (WVU) have demonstrated the ability to build a burn pit generator with the capacity to burn and smolder standardized waste with JAA fuel, Fig. 3A. The burn pit emissions generated are then delivered to freely moving rodents in an inhalation chamber, Fig. 3B. Emissions delivered to the exposure chamber can achieve particle concentrations of 0.5–50 mg/m^3^, and extensive characterization is possible throughout exposures. Aerosols are sampled for subsequent assays and characterized in real time via numerous ports in the exposure chamber. Examples of emission characterization data are shown in Fig. 4. The complexities of emission components and delivery methods highlight the challenges in using preclinical models to accurately replicate and study the health effects of burn pit exposures, underscoring the need for further refinement and validation of these models to better understand their implications for human health.

To date, rodent studies have identified inflammatory responses and respiratory dysfunctions following exposure to PM and other toxic components prevalent in burn pit smoke [15]. Pulmonary diseases and the onset of inflammatory responses have emerged as major health concerns in Veterans, and the majority of preclinical modeling studies have focused on pulmonary impacts [15, 67]. Observations from various models have shown both acute and chronic inflammatory responses in mice exposed to simulated burn pit emissions, resulting in increased lung resistance and changes in inflammatory cytokines [15, 45, 67, 69, 71]. A common finding across multiple models is the elevation of inflammatory markers, such as IL-2, IL-4, IL-5, IL-6 IL-13, and TNF-alpha, suggesting a consistent immune response to the simulated burn pit exposures [62, 67, 69, 70]. Not all studies observed significant histopathological changes in the lungs, which could be due to varying exposure durations, compositions or concentrations [62, 68]. The extent of physical changes in lung tissue varied significantly among studies, with some reporting minimal to no changes and others noting increased mucin staining and goblet cell hyperplasia, indicative of mucous membrane irritation and potential respiratory dysfunction [68, 71]. These results derive from simple to complex mixtures, different delivery methods, and duration of exposure [15, 53, 62, 67–71]. Further experimental design aspects include incorporating environmental or physiological stress to mimic the military environment [15].

Cumulative knowledge gained from preclinical models needs to take into account the various parameters of the model system used. It is likely that multiple approaches will be needed to fully capture the range of pathobiologies reported following exposures. As progress is made in establishing how to model specific disease phenotypes, the field will move forward using these pre-clinical models to test treatments, contributing to the understanding of how burn pit exposure affects health.

## Conclusions

The PACT Act has significantly impacted Veteran health and welfare, with over 5 million Veterans screened for burn pit and military toxic exposures by the end of 2023 [48]. Among these, 2.1 million Veterans reported at least one toxic exposure, with respiratory complaints such as allergic rhinitis, maxillary sinusitis, bronchial asthma, and constrictive bronchiolitis being the most frequently reported health concerns [48, 74]. Other health conditions that may emerge or increase over time include gastrointestinal, urological, autoimmune, chronic multi-symptom illnesses, toxic brain injury, and various malignancies [16, 45, 46]. Despite these screenings, there is currently a lack of comprehensive data directly linking the prevalence of specific respiratory diseases, such as shortness of breath or airways/interstitial lung disease, to these exposures. Existing data sources, including the VA Airborne Hazards and Open Burn Pit Registry and various epidemiological studies, often focus on overall symptom reporting rather than detailed diagnoses across large cohorts.

Estimating the fraction of Veterans with specific respiratory conditions remains challenging due to several factors. First, the heterogeneity of exposures where Veterans have encountered a wide range of toxicants in varying types and levels affects the risk and severity of respiratory conditions, making it difficult to generalize findings from smaller studies to the broader population of exposed Veterans [46, 75]. Additionally, variability in diagnostic criteria and reporting across studies, ranging from self-reported symptoms to biopsy-confirmed diagnoses, leads to inconsistencies in data, complicating efforts to calculate an accurate fraction of affected Veterans. Underreporting is also a concern, as many Veterans may not seek medical evaluation or may present with non-specific symptoms that are not immediately linked to toxic exposures, further affecting data accuracy.

Moving forward, there is a critical need to refine our understanding of these exposures and their associated health outcomes by developing well-designed research studies and robust models to capture the complex interactions of burn pit exposures and their effects on health [15, 75]. Collaborative efforts among the VHA, National Institutes of Health, Department of Defense, academic institutions, and private entities have led to the creation of valuable databases, advanced technologies, and extensive sample repositories. Effectively utilizing these resources and engaging the right experts for collaboration is essential to advance both preclinical and clinical research. Establishing standardized protocols for preclinical modeling of military toxic exposures would facilitate cross-study comparisons in exploring disease development and progression.

There is a need to identify reliable diagnostic and pathological measures for disease-relevant biomarkers to move toward evidence-based therapeutics. Understanding exposure-induced pathologies, defining disease endotypes, and bridging preclinical and clinical biomarkers are important next steps. For small airway diseases, in particular, significant gaps remain in diagnostic measures and pharmacotherapies. Innovations in non-invasive functional lung imaging and remote monitoring technologies hold promise for improving health assessments, disease diagnosis, and treatment outcomes. Moreover, integrating genomic, proteomic, and pathomic data into research and clinical management will be vital for personalizing care and improving outcomes for exposed Veterans.

Air pollution is a global health challenge and a leading cause of early death, contributing to lung cancer development even among non-smokers [25]. Wildfires, which generate high concentrations of toxic particulate matter from burning biomass and man-made materials, affect both firefighters and the general population by inducing a range of negative systemic health effects [76, 77]. Insights from the diverse extra-pulmonary biological effects resulting from air pollution exposures are reshaping our understanding of the multifaceted impacts of burn pit exposures and their potential systemic consequences. The close links between military toxic exposures, air pollution, and occupational exposures underscore broad implications for both Veteran and general population health. Future studies on military exposures have the potential to benefit not only Veterans but also public health more broadly.

## Data Availability

No datasets were generated or analysed during the current study.
